# Evaluating medical student engagement during virtual patient simulations: a sequential, mixed methods study

**DOI:** 10.1186/s12909-016-0530-7

**Published:** 2016-01-16

**Authors:** Lise McCoy, Robin K. Pettit, Joy H. Lewis, J. Aaron Allgood, Curt Bay, Frederic N. Schwartz

**Affiliations:** A.T. Still University, School of Osteopathic Medicine in Arizona, 5850 E. Still Circle, Mesa, AZ 85206 USA; A.T. Still University, Arizona School of Health Sciences, 5850 East Still Circle, Mesa, 85206 Arizona USA

**Keywords:** Engagement, Virtual patient simulation, Learning-centered instruction, Technology-enhanced learning

## Abstract

**Background:**

Student engagement is an important domain for medical education, however, it is difficult to quantify. The goal of this study was to investigate the utility of virtual patient simulations (VPS) for increasing medical student engagement. Our aims were specifically to investigate how and to what extent the VPS foster student engagement. This study took place at A.T. Still University School of Osteopathic Medicine in Arizona (ATSU-SOMA), in the USA.

**Methods:**

First year medical students (*n* = 108) worked in teams to complete a series of four in-class virtual patient case studies. Student engagement was measured, defined as flow, interest, and relevance. These dimensions were measured using four data collection instruments: researcher observations, classroom photographs, tutor feedback, and an electronic exit survey. Qualitative data were analyzed using a grounded theory approach.

**Results:**

Triangulation of findings between the four data sources indicate that VPS foster engagement in three facets:

1. Flow. In general, students enjoyed the activities, and were absorbed in the task at hand.
2. Interest. Students demonstrated interest in the activities, as evidenced by enjoyment, active discussion, and humor. Students remarked upon elements that caused cognitive dissonance: excessive text and classroom noise generated by multi-media and peer conversations.
3. Relevance. VPS were relevant, in terms of situational clinical practice, exam preparation, and obtaining concrete feedback on clinical decisions.

**Conclusions:**

Researchers successfully introduced a new learning platform into the medical school curriculum. The data collected during this study were also used to improve new learning modules and techniques associated with implementing them in the classroom. Results of this study assert that virtual patient simulations foster engagement in terms of flow, relevance, and interest.

**Electronic supplementary material:**

The online version of this article (doi:10.1186/s12909-016-0530-7) contains supplementary material, which is available to authorized users.

## Background

Undergraduate medical education is moving toward technology-enhanced, engaging, experiential learning [1–3]. Educators concur that active, learner-centered instructional approaches are more successful than lecture for capturing the interest of the present generation of learners [4–7]. New media literacy research [8, 9] indicates that the current Internet generation student population is more fully engaged by electronic media and audio-visual stimulation. In response to these trends, we sought to increase student focus, participation, and collaboration through interactive, technology-enhanced instruction during first year small group clinical case practice [6, 7]. Following a student-centered andragogy [10] calling for increased use of education technology, active learning, student choice, swift feedback, and peer discussion, we designed interactive virtual patient simulations (VPS) to increase engagement.

VPS are interactive computer simulations of real-life clinical scenarios for the purpose of medical training, education, or assessment [11]. This medium was selected because VPS deliver instruction in an interactive modality suitable for first year medical students, who are typically comfortable with multi-media and web-based games [8, 12–14]. Saleh [15] asserts VPS provide students with control over the learning episode and opportunities to learn by trial and error. VPS provide a risk free environment for practicing clinical encounters [16]. Situational learning theory [17] supported our efforts to foster peer co-construction of knowledge by solving real life problems in a situational context [17] (a patient encounter) through the medium of a VPS.

Prior to this project, small group cases were delivered via PowerPoint. Similar issues reported by other medical educators [18] were observed: during small groups, some students did not have a chance to contribute to discussion as much as others, self-assessment was not part of the lesson plan, nor did students have a chance to reflect upon what they had learned about each case. Congruent with design recommendations of simulation game educators [19–21] and VPS experts, this project sought to enliven the student experience through a participatory experience inviting clinical discussion, self-guided interaction, and reflection.

Education game theorists suggest that virtual worlds provide learning spaces in which pleasure and satisfaction are derived from increased competence [13, 22], and that virtual simulations are effective because they provide students with learning spaces, which allow them freedom to experiment, process evidence, and collaborate in authentic scenarios [23]. It was hoped that the new VPS would draw students into the patient’s story, contextualized in a health center clinic environment. Deterding et al. [22] describe the activation of learner self-motivation through autonomous goal pursuit, rule negotiation and symbolic reasoning, leading to creativity. Rotgans and Schmidt [24] described engagement while problem solving as “situational interest”, a state supported by peer inquiry, in which student curiosity is piqued by the enigmatic nature of a problem as they proceed to search for data to answer questions.

While several studies have documented the effectiveness of VPS in medical education [16, 25, 26], few studies apply mixed methods to measure medical student engagement during VPS. Edelbring et al. [18] conducted a phenomenological, mixed methods study featuring recorded interviews with 31 medical students. Results from this study indicated that the VPS allowed students to actualize theoretical knowledge, and were characterized by student’s active engagement in reasoning. Pantziaras et al. [27] studied the perceptions of eight students and nine faculty after a VPS module, and reported that participants received the exercise well in terms of realism, excitement, engagement, and active learning. Courteille et al. [28] collected open-answer comments from 68 fourth-year medical students after a computer simulation. These researchers found students appreciated the immediate feedback received after clinical decision-making, and that outcomes related to arriving at a correct diagnosis after 10 min during a VPS Objective Structured Clinical Examination (OSCE) were correlated with the process of thinking aloud during the exercise.

### The construct of engagement

In this study, we explore aspects of engagement in the context of simulated clinical decision making for first-year medical students. Our VPS provided case scenarios that required rapid-fire group clinical decisions during a timed exercise. For the purpose of our research, we measured engagement as flow, relevance and interest.

#### Flow

Flow is a construct used by education researchers to operationalize facets of engagement and active learning [29–31]. According to Csíkszentmihályi [32], flow experience is facilitated by clear objectives, immediate feedback, the proper level of lesson difficulty, and control. Flow manifests as increased concentration and focus, transformation of time, and a perception that an activity has intrinsic value.

Schiefele and Raabe [31] assert that engagement may be measured by a participant’s self-reported degree of flow (absorption in the activity), and *state concentration* (concentration on task). Admiraal et al. [29] explain that flow invokes the “growth principle” (p.1186). Once students master a task, they seek a progressively more challenging task. Even demanding intellectual activities can promote flow because these activities provide satisfying interactions that scaffold students through a series of difficult tasks. Furthermore, these researchers explain flow may be measured in two ways: by self-assessment, meaning the students fill out a survey, or by instructor observation of a learning experience.

#### Relevance

Learner motivation experts [17, 30, 33] explain that adults are more intrinsically motivated to complete learning tasks when they understand their full value and relevance to academic, workplace, or personal goals. We sought to understand whether VPS lessons would be deemed valuable in terms of a lesson’s ability to increase interest in clinical practice, provide relevant feedback, and enhance preparation for examinations.

#### Interest

These VPS were designed to add variety [5] to the learning environment and provide exposure to new experiences in terms of patient encounters.

### Research questions

The aim of this study was to test a new type of VPS intended to foster engagement during clinical case practice. This study investigated two research questions:

1. In which ways do VPS foster engagement?
2. How can these activities be improved next implementation?

## Methods

### Research design

Authors selected a mixed methods design to improve the overall strength of the findings, incorporating both qualitative and quantitative data [34]. To answer the research questions, researcher observations, classroom photographs, tutor feedback, and student Exit Survey results were triangulated. This process provided a rich set of data with which to describe specific mechanisms whereby VPS engaged students during simulated patient encounters. Since data collection progressed over four sequential classroom sessions, culminating in a student exit survey at the end of the year, we describe this study as “sequential”. A modified design-based research (DBR) approach was used to gradually improve the new learning tools over time [35]. The expected outcome was that the VPS would be engaging. Following methods used by DBR experts Barab and Squire [35], and grounded theory processes similar to those used by Bateman et al. [1], data were reviewed in order to identify areas for improvement, resulting in suggested “design solutions”.

### Setting

This design-based research study took place at an osteopathic medical school in the Southwest United States. Subsequent to a field test in August 2013 with the previous cohort, the main study took place October - December, 2013. This intervention–VPS modules–was implemented four times over two months (with the entire sample of student participants participating in each implementation). Data collection occurred during the neuro-musculoskeletal (NMSK) and cardio-pulmonary courses as part of weekly mandatory small group class meetings for first-year students. This study was exempted by two university ethics committees from ongoing reporting requirements for “human subjects” research: 1) the A.T. Still University Institutional Review Board and 2) the Arizona State University Office of Research Integrity and Assurance.

### Participants

The entire class of 2017 first year medical students (n = 108) participated in the study as part of normal classroom activities. Six faculty small group tutors participated as part of normal teaching duties. Tutors were DO and MD physicians in the disciplines of family medicine, internal medicine, neurology, and pediatrics, with 5–40 years of primary care practice, and 1–15 years of experience leading small groups.

### The intervention

**Fig. 1:**
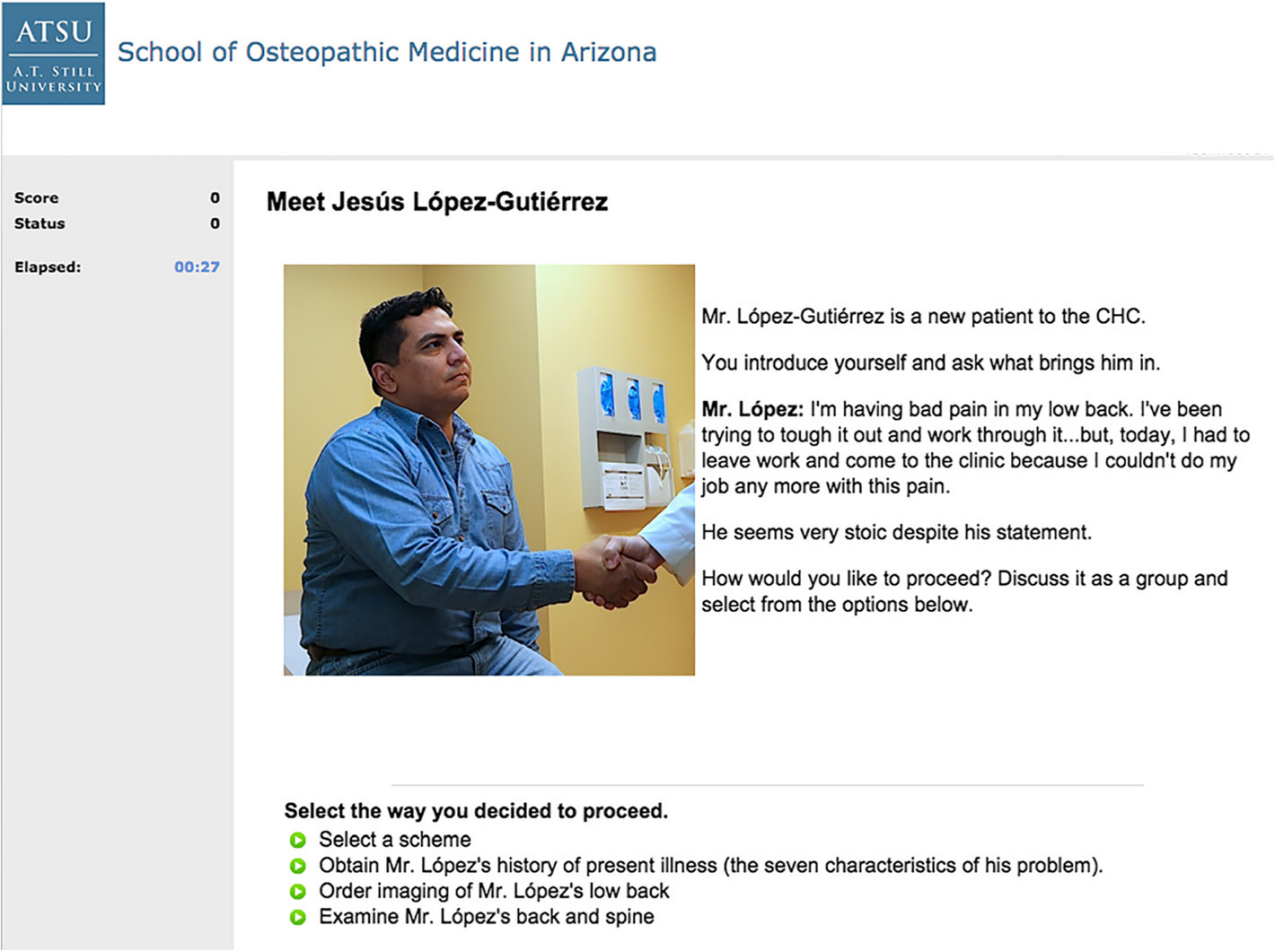
A sample page from a VPS. An image of an actor playing a patient. Printed with permission from the actor

In 2013, the school’s Technology-Enhanced Active Learning for Medical Education (TEAL-MEd) team [36] faculty developed four VPS for the current study using the clinical presentation approach [37, 38]. A screen capture from a sample VPS case is provided in Fig. 1.

These VPS exercises afforded student teams the opportunity to manage a patient encounter and formulate a general diagnosis. The modules prompt students, working in teams of three, to make clinical decisions through every phase of the clinical encounter (presentation, history, physical exam, lab findings, diagnosis, treatment, and plan). Consistent with VPS design principles suggested by experts such as Posel, McGee and Feiszer [39], these exercises were designed to engage students through peer discussion regarding each clinical choice, and to provide continuous, immediate feedback after every decision so that students have the opportunity to learn from errors. Consonant with design principles suggested by Huwendiek et al. [40] the modules were designed to be 1) relevant in terms of being important cases, 2) level-appropriate, 3) interactive, 4) offer concrete feedback for each decision, 5) include sound and video media, 6) focus on relevant learning points, 7) summarize key learning points, 8) provide the authentic experience of being the lead healthcare provider (doctor in charge), 9) require students to make all the clinical decisions a physician would make, and 10) enhance clinical reasoning (in our VPS format, students practice inductive reasoning based on synthesis of evidence).

Modules were developed using the DecisionSim™ [41] system, which provides decision dilemmas to students through a web-based case player. We selected this format for its branching design, ease of use and multimedia capabilities. The case-writing template allowed faculty authors to embed video, sound, and links to external sources. As described using the typology framework suggested by Huwendiek et al. [42], (See Additional file 1), the VPS were designed for first year students, followed a progressive disclosure approach beginning with a clinical presentation. The modules aligned to both clinical and professionalism objectives, and provided pre-formulated feedback for every clinical choice. The VPS modules were outfitted with gamification elements [22], including meters for score and status (health status of the patient), cost of care, dramatic story line, rewards for high scores such as videos, and auto-play sound and video. The VPS modules tracked learner performance; student teams earned points for each clinical decision during the case. The maximum number of points teams could attain for a given case was 100 points.

### The technique

During the NMSK course, students engage in case practice once every week. Due to small group classroom space constraints, first year students are divided into two sessions for case practice. During Session 1, half of the student sample (*n* = 54), was distributed to six break-out rooms, each supported by a physician-tutor, for a two-hour case practice with three cases (the first of which was a VPS, the remaining two were Powerpoint cases). During Session 1, the other half of the student sample (*n* = 54) attended Anatomy lab in a different classroom. During Session 2, the activities were reversed. To clearly frame the VPS lesson, tutors handed out instructions to students at the beginning of the class period, a technique that reflects lesson design suggestions by Winberg and Hedman [43]: providing guiding instructions at the beginning of a computer simulation is correlated with challenge, enjoyment, and concentration.

### Lesson sequence

**Fig. 2:**
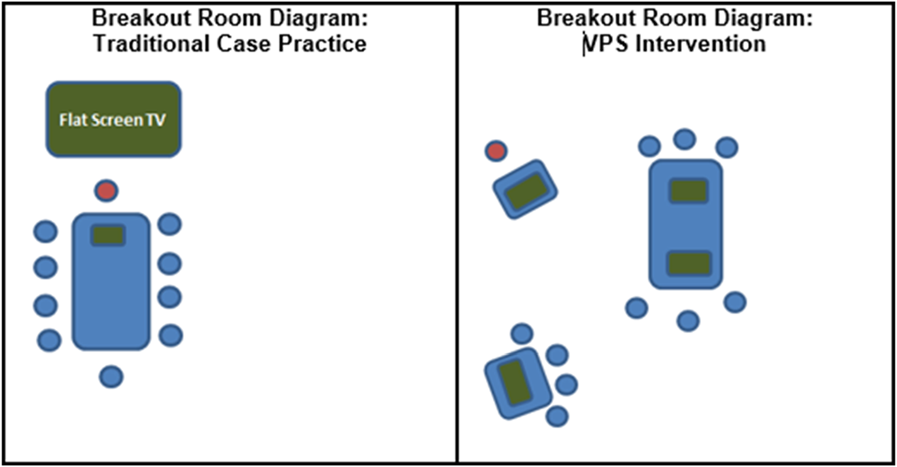
Small group room configurations for sessions 1–4. A diagram contrasting the difference in configuration between traditional small group (left) and sessions including VPS (right). Blue dots represent first year medical students, red dots represent the tutors (clinical faculty), and green rectangles represent computer screens

At the request of tutors, students quickly formed self-prescribed peer-groups with three-to-four members. Sharing one laptop, each team assumed control of an exercise and worked through the 20-min DecisionSim VPS case. Tutors circulated in the small group room. After completion of the case study, each tutor led a debriefing of the VPS case. Next, tutors continued with their presentation of a traditional case (in PowerPoint). Figure 2 contrasts the breakout room configurations for traditional and intervention formats. During VPS, students moved into the intervention format, and during the traditional part of the lesson, they moved back into traditional formation.

### Measures

This study reports data from four measures: Researcher Observations and Analysis Memos, Classroom Photographs, Tutor Feedback and Exit Survey. Results are reported separately by data source, and then triangulated to answer each research question or hypothesis.

#### Measure 1: observation form and analysis memo

Observation during VPS activities served two purposes: to document whether sessions were implemented as planned (with fidelity) and to identify facets of the lesson plan to improve. The observation form is available in Additional file 2. The grounded theory process [44] entailed reading observation notes, then using them as a resource to generate a more subjective researcher analysis memo. This method prompts a researcher to reflect deeply upon the classroom dynamics during the learning activity.

Observation data were processed and analyzed using grounded theory protocols outlined by Corbin and Strauss [44] as follows:

- Reading through the transcribed responses sentence by sentence to open code concepts such as *involvement*.
- Creating separate codes to indicate variation in the dimension of the code, For example, if a code is *enthusiastic outburst*, an opposite might be *complaint*.
- Organizing the codes into wider categories, such as *flow*.
- Requesting colleagues to review the codes and categories.
- Developing a code book which defines the scope of each code.
- Using graphic organizers to compile quotations from raw data into generalized descriptions and illustrative examples.

To strengthen analysis, allow for data mixing, and provide an audit trail, MS Word documents with narrative data such as researcher analysis memos and tutor feedback were uploaded to HyperResearch™3.5.2. This software allows a researcher to tag segments of text using an open coding process [44]. The next step was to associate the codes with the *a priori* domain, engagement. HyperResearch-generated summary reports listing codes and data sources were exported into Excel spreadsheets.

Data were sorted by VPS activity, transition, or traditional modes of instruction. Transition is defined as the period of time when some student teams were finishing a VPS, while other students, having already finished a VPS module, were waiting for the next segment of a lesson: traditional small group instruction led by a tutor. Observation data were processed and analyzed using grounded theory protocols outlined by Corbin and Strauss [44] as follows: the frequency of codes (data instances) was tabulated, aggregating across the four dates of implementation.

#### Measure 2: classroom photographs

Photographic data provide additional evidence of engagement during classroom activities. Authors attempted to document the *design case* (e.g., implementation of the novel lesson design) with a rich digital record of the event happening simultaneously in six concurrent classrooms. In collecting digital records, an attempt was made to protect the privacy of subjects. On the first day of the study, tutors provided a printed explanation of the study that pre-informed the students they would be video-recorded and photographed. This form explained that digital images that could identify participants would not be shared in a publication format without their express written consent.

**Fig. 3:**
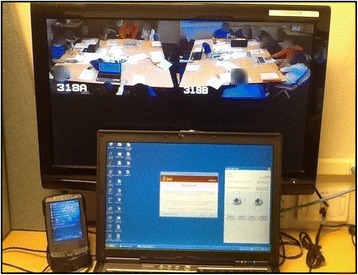
A control room classroom observation station. Printed with permission from SOMA. In this photograph, student and instructor faces were intentionally blurred to obscure identities

We used a pragmatic, systematic approach suggested by visual ethnographer Sarah Pink [45] to collect photographic data. A randomized approach involved capturing a round of photographs at 10 min intervals with an iPad (for 30 minutes of team practice with VPS and 30 minutes of traditional small group instruction). Figure 3 is a view of the observation station in the small group control room.

Classroom photographs were analyzed as an additional data point to increase the validity of the findings. Pink [45] explains that “a realist approach” (p. 124) to coding complex visual data offers a crucial means of managing data and triangulating findings. In conducting video research, Derry et al. [46] advise researchers to address the following four challenge–in each case we describe how we specifically addressed the challenges:

- o Selection/Sampling: In our study, all classrooms were video recorded for one hour each. A specific regimen for sampling photographs from these videos was outlined.
- o Analysis: We describe the process of coding and analysis of photographs; e.g. grounded theory [44], quantified using a rubric.
- o Technology: The school’s remote video system Arcadia^TM^ was used for observation of classroom sessions.
- o Ethics: Students and faculty were informed prior to being video recorded. Students provided written consent for photographs displayed in conference presentations and publications.

To our knowledge, using video observation to measure team dynamics while a class is actively participating in healthcare VPS is a rarely-attempted technique. Courteille et al. [28] video-recorded a sampling of VPS OSCE’s. Upon review of session recordings, these researchers categorized participant behavior patterns using a rubric measuring the constructs of student-assistant interaction, expression of uncertainty, stress, flow, mouse handling, and keyboard handling.

Photographic data analysis for the current study unfolded in three steps. After downloading 292 video images from the iPad, image data were stratified into electronic folders for each collection date by sequence and modality of instruction: VPS, transition, or traditional small group instruction. The photographs were analyzed using a coding worksheet, in lieu of the HyperResearch database, as it was inefficient to store photographs on this system. In the digital archive, each photograph was labeled with a discrete numeric designation. During the process of open coding, each photograph was referenced by numeric designation.

Next, the entire library of photographs was open-coded to discover themes [45]. To mitigate subjectivity, code descriptions were developed pragmatically, based upon body behavior, such as leaning in. Codes grouped into categories and themes mapped to the *a priori* domain, engagement: *highly engaged*, *engaged and leaning in*, *interactive, focused on task*, *transition activities*, *passive listening*, *low enthusiasm*, and *closed.*

Literature on interpreting photographs to analyze medical school small group engagement is non-existent to date. Authors looked to other disciplines to obtain insight in developing an analysis rubric. Middendorf and McNary [47] developed a classroom observation rubric for observing social science faculty using video review of classroom interactions. Riley-Tillman, Methe, and Weegar [48] rated class-wide engagement of elementary school children, and observed whether students were on task by using an observation protocol and check marks for on task behavior. Active engagement was defined as participating by raising a hand, writing, or talking about a lesson. Passive behavior included listening to the teacher, reading silently, or looking at instructional materials. Literature from the team-based learning for healthcare professions [49] describes body language as positive and engaged when students are leaning in, communicating effectively, and not engaging in off-task behaviors such as checking email. Westberg and Jason [50] describe the non-interactive student behavior during authoritarian small group participation as “distant and guarded” (p. 20). Additional literature on the interpretation of body language arises from the business fields in relation to corporate meetings [51, 52]. This body of popular wisdom concludes that leaning in, taking notes, and facing the team denotes engagement, while body language such as crossing the arms, slumping down or leaning on the table indicates less energy or lack of enthusiasm. Other cognitive scientists such as Cuddy [53] remark that when meeting participants are huddled down, this indicates a less powerful stance, and when they are stretched out, they are confident. Using this body of theory as a basis, the authors reviewed the slide shows of the photograph data, developed a list of initial codes, and reviewed them with the TEAL-MEd committee to assess the range of codes to ensure none overlapped and all were defensible. They suggested using codes toward objective categorization of physical body movements.

#### Measure 3: tutor feedback form

Six tutors facilitated four VPS activities, each overseeing approximately 10 students. The tutor feedback form is available in Additional file 3. The process of analyzing tutor feedback began with reading through anonymous comments and typing each comment into a compilation text document. In between simulation sessions, the TEAL-MEd committee met to discuss the de-identified tutor feedback to determine whether there were technology glitches or other issues to address. Tutor feedback data were coded and analyzed using HyperResearch software.

#### Measure 4: exit survey

The Exit Survey instrument was developed by McCoy [54] and validated through a stepwise process. First, literature searches were conducted to review surveys related to VPS and video games for medical education simulations [14, 24, 31, 43]. These searches revealed no published survey instruments that interrogated the complete range of topics associated with this study and specific educational context. This process rendered an original survey instrument refined through several iterations of implementation and peer review. The original survey was field tested in the spring of 2013, and expanded by eight items for a final 28-item survey (Additional file 4). Eight items of this survey related to an engagement sub-scale (Table 1).

**Table 1:** Survey items: engagement

Sub-construct	Specific attribute	Source reference
Flow	Time awareness	Schiefele & Raabe, 2011 [31]
Flow	Enjoyment of working on tasks	Schiefele & Raabe, 2011 [31]
Flow	Absorption in activity	Schiefele & Raabe, 2011 [31]
Flow	Exciting tasks	Schiefele & Raabe, 2011 [31]
Relevance	Increased interest in clinical practice	De Bilde et al. 2011 [33]
Relevance	Provided relevant feedback	De Bilde et al. 2011 [33]
Interest	Provided exposure to new experiences	Prince, 2004 [5]
Interest	Added variety	Prince, 2004 [5]

The exit survey contained an introductory paragraph informing participants that responses would remain anonymous and data would be aggregated. The first item of the survey gave students the option to exclude their responses from analysis and results were filtered to remove the responses of participants who chose this option. Survey responses were downloaded from SurveyMonkey™ as Excel spreadsheets. SPSS^TM^ version 22 (IBM Corp, Armonk NY) was used to estimate inter-item reliability.

## Results

### Researcher observations and analysis memos

Researcher observation notes and memos indicate that VPS foster engagement. These data sources rendered insights regarding eight aspects of engagement: anxiety, focus, humor, interest, enthusiasm regarding the score, gratitude to the case author and wish to continue. Table 2 provides examples of data for each of the eight aspects.

**Table 2:** Observation notes and memos by domain: engagement

Code	Sample researcher analysis memos regarding VPS activities
Anxiety	*[First case, first five minutes]. “A student expresses that she feels anxious to complete this case: ‘we are being video-recorded, I don’t know what to do about the competency task, it’s difficult, there is time pressure, and there is no professor here.’”*
Note-Taking	*“Very few students were observed taking notes during VPS.”*
Focus	*“In the small group room I observed, the students were fully engaged and at one point, even stopped looking at the game [VPS] and sat in a triangle discussing the case.”*
Interest	*“One colleague observing from the control room said the students were “pimping” each other” [this means challenging each other with questions about the case].”*
Humor	*“[In reviewing action on monitors from the control room].” There was laughter in several rooms.”*
Enthusiasm about score	*“Students discuss their score at the end of the case (out of 100 points) 85 %. Not bad!”*
Gratitude to professor	*“At the end of the case, the students thank the absent professor who wrote the case: ‘Thanks, Dr. C!’”*
Wish to continue	*“Students asked to stop at 20 min requested more time to do the case.”*

Just prior to the first VPS case, one student expressed apprehension about the exercise. This was the first time she had encountered a decision simulation, and was unfamiliar with the user interface. She did not know how to pace herself. While cognizant of a time limit of 20 minutes, the students in her group worked through the case successfully, despite these initial concerns.

Students displayed attention and focus during each case observed. In one instance, team members were so drawn into the case they forgot time and task, and sat in a triangle, avidly discussing case details. Students demonstrated an interest in the cases by working above and beyond arriving at diagnoses: they quizzed each other through the case objectives, defined terms for each other, and tested each other’s knowledge regarding key concepts.

In one of the four cases, one student was quite reserved and aloof at the beginning. Gradually, she was drawn into the epicenter of the discussion. Several instances of student laughter and humor during VPS sessions were noted. This observation was corroborated by other observer colleagues. On more than one occasion, students expressed interest in their scores, and enthusiasm upon receiving a high score. At the close of one case, students politely thanked the professor who wrote the case (though he was not present in person).

For the first case, tutors were instructed to end a VPS case after 20 min, which they did. Some student teams seemed reluctant to end the session, and requested more time to complete the case. Subsequently, the small group tutors requested there be the option to continue longer with the simulation cases. Students were not graded on this activity and were not required to continue. However, as observed frequently, students continued with the case to review topics such as treatments and clinical pearls from the virtual clinician mentor, summarizing key points to remember.

### Tutor feedback

Six tutors provided 28 feedback forms. The tutor feedback forms solicited commentary in open answer format. Feedback was divided into *feedback on the learning experience* (Table 3) and *feedback on the VPS modality.* Tutor feedback statements related to domain engagement are reported in Table 3. Of the 16 engagement statements collected, two indicated a lack of engagement.

**Table 3:** Tutor feedback regarding engagement

Theme	Code/ frequency of code	Tutor feedback
Flow	1-Attention	*“Good attention.”*
		*“Good attention and flow.”*
	5-Involvement	*“Very quiet but everyone involved.”*
		*“All: Good immersion. Discussions good depth.”*
		*“Very engaged.”*
		*“Involved and immersed.”*
		*“Good student interaction and participation.”*
		*“Good involvement.”*
	5-Engagement	*“Seemed engaged.”*
		*“3 groups. 3 all engaged.”*
		*“Had fun and learned.”*
		*“8 out of 9 students engaged.”*
		*“One group enjoyed this more.”*
	2-Enthusiastic outburst	*“Some good excitement noted. Also: WHAT?!”*
		*“Some cheering noted and arm waving.”*
	1-Wish to continue working	*“During the remainder of the lesson, some students were sneaking back onto DecisionSim to finish the case.”*
	1-Not in the flow	*“Students finished case very quickly. Discussions were brief.”*

Tutors made several positive comments about the learning activity, including *Good immersion*, *Discussions good depth,* and *Very engaged*. Their feedback indicated that the activity held the attention of the majority of the students. Some enthusiasm was noted. For example, at times, students were cheering when they chose correct answers. One tutor noted that during traditional case instruction during a subsequent case, some students returned to the simulation case. In terms of lack of being ‘in the flow’, one tutor mentioned that during one case, students discussed little and finished the case quickly.

### Classroom photographs

In the process of implementing the VPS, 292 photographs were screen-captured during four VPS activities from control room video images. Table 4 reports the numbers of photographs taken for each of the four activity implementations. The data set included a proportionally greater number of VPS photographs (165) because the small group tutors allowed the students to continue with the VPS beyond the 20 min time limit. Transition time photographs reflect activities that were happening in between VPS and traditional instruction led by the tutor via PowerPoint. During the transition interval, students were chatting, reviewing on their own individual laptops, or silently waiting for other classmates to finish the virtual simulation.

**Table 4:** An inventory of classroom photographs by modality of instruction

	VPS	Transition	Traditional	Total
Activity 1	49	2	30	81
Activity 2	45	10	30	85
Activity 3	41	9	10	60
Activity 4	30	13	23	66
Totals	165	34	93	292

Table 5 summarizes the entire data set of 292 photographs categorized by code/theme, and engagement level. The photographic data in Table 5 suggest that during VPS, students displayed rapt concentration, leaning in, focusing on task, and interactive behaviors. Traditional instruction resulted in frequent instances of passive listening or low enthusiasm.

**Table 5:** Classroom photographs by level of engagement and modality of instruction

Level	Level of engagement	Category/code	VPS	Transition	Traditional
		Total photographs taken	Photo *n* = 165	Photo *n* = 34	Photo *n* = 93
High Engagement	Very High	Highly Engaged— at least one student	37	4	1
		Pleased expression			
		Rapt concentration			
	High	Leaning In—all students	51		
	High	Focused on Task – all students in the team	64		22
	High	Interactive-at least one student	42	6	11
		Gesturing to illustrate joint			
		Pointing to the simulation on laptop			
		Discussing with tutor			
		Taking notes			
		Discussing with peers			
Medium	Medium	Transition Activities^a^		34	
		Some teams finishing the simulation			
		Completing Competency Task			
		Waiting while others finish VPS			
		Discussing with tutor			
	Medium	Passive Pose— all students			25
Low	Low	Low Enthusiasm but Focused on Task: at least one student	13		22
		Head in hand leaning down			
		Lethargic demeanor			
	Low	Low Enthusiasm-Less Focused on Task: at least one student	5		13
		Not paying attention to PPT			
		Difficult to see VPS screen			
		Leaning away			
	Very Low	Reserved: at least one student			21
		Sitting with arms crossed.			

Student N = 107. Photo N = 292. Some photographs were cross-coded to more than one category

^a^There was a period of transition between VPS and traditional small group instruction

**Fig. 4:**
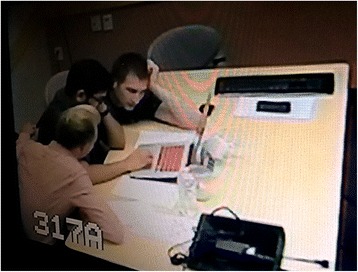
A team of medical students completing a VPS. Classroom photograph, printed with permission from the three students depicted

In Fig. 4, three students lean into the lesson, showing a state of high engagement by focusing intently on solving the patient case.

### Exit survey

After experiencing four VPS, 105 students participated in an electronic survey during class hours; the response rate was 97 %. Respondents reported their ages as 20–25 (67.6 %), 26–30 (26.7 %), and 31–35 (5.7 %). Item analysis revealed all items contributed to the survey. The Cronbach’s α for the Engagement scale was (α = .882), participant *n* = 103, item *n* = 8.

By collapsing two of the five-point Likert scale response categories into *agree* vs. *disagree* and ordering items in terms of student agreement, it was easier to evaluate the aspects of the VPS rated most highly by students. The survey instrument measured impressions of engagement with four related sub-items regarding variety of learning modality, new experiences, increased interest in clinical practice, and the relevance of the feedback gained during VPS.

The first sub-component of engagement measured was flow, or "*absorption in task*” Attributes of flow include unconscious passage of time, enjoyment, and excitement of task. Table 6 displays the results for survey items related to the flow aspect of engagement, designed with Likert items validated by Schiefele and Raabe [31].

**Table 6:** The value of virtual patient simulations in terms of flow

Statement	Strongly agree/agree
	*N (%)*
I enjoyed working on the tasks.	89 (85.6)
I did not realize how time passed.	70 (67.3)
I found the tasks to be quite exciting.	66 (63.5)
I was completely absorbed in the activity.	63 (60.6)

*N* = 104

In terms of flow, students rated enjoyment of working on the tasks most highly. Statements regarding loss of time awareness, excitement and self-perception of absorption in the activity were rated highly by nearly two-thirds of the participants.

Two additional facets of engagement measured with the exit survey were interest and relevance. Table 7 reports the student ratings with regard to these facets. Respondents rated interest and relevance aspects of VPS highly, with *variety* receiving the highest ratings.

**Table 7:** The value of virtual patient simulations in terms of interest and relevance

Statement:	*N(%)*
VPS added variety to the learning environment.	97 (92.4)
VPS provided exposure to new experiences.	82 (78.8)
VPS provided relevant feedback.	82 (78.1)
VPS increased my interest in clinical practice.	75 (71.5)

*N* = 105

### Exit survey open responses

Student participants provided 55 open responses to the last item of the electronic survey: *How may we improve these [VPS] activities*? Statements were downloaded from SurveyMonkey, and open coded into eight themes as shown in Table 8.

**Table 8:** Exit survey open comments by theme and frequency

Theme	*n*	Frequency/code	Sample student comments
General Comment	18	4 no improvements to recommend	Facilitator discussions *“Facilitator discussions are way better (but it depends a lot on which facilitator).”*
		1 sims were good activities	
		1 refining the sims	
		1 not sure they are effective	
		1 facilitator discussions	
Activity Format	14	6 triads should meet in separate rooms	Triads should meet in separate rooms. *“Videos are a nice idea but when several groups are together in the same room we couldn’t really watch the videos or if we did the sound would overlap with each other so a lot of times we didn’t even bother to watch the videos even though they looked informative and interesting.”*
		5 when to schedule the sims	
		1 add complexity to the activity worksheet	
		1 would’ve rather picked my group	
		1 students should prepare prior to the simulation	
Desire for Individual Study	4	3 bank of simulation situations	Bank of Simulation Situations. *“Eventually, if you’re able to develop a bank of simulation situations, I think that would be really helpful. I would practice them in my own time.”*
		1 independent study	
Case Content	6	2 review material too extensive	Review Material Side Loops *“There were so many asides when all I wanted to do was assess and treat my patient. I didn’t want to learn about each aspect of care as I made decisions. I wanted to assess, think quickly, treat, and then find out what happened—kind of like a video game.”*
		1 increase the complexity of the cases	
		1 match case content to large group lesson	
		1 tasks too detailed	
		1 diagnosis feedback	
			Diagnosis Feedback *“Go through the scenario and then provide relative information on why certain diagnoses are correct. More pertinent negatives would also be helpful. In other words, I want to know why certain diagnoses are wrong.”*
Case Format	6	2 embedded videos	Patient Chart *“Have the option to go back and look at the HPI* ^*a*^ *and previous screens in case we forgot what the age and specific symptoms of the patient was.”*
		2 patient chart	
		1 length of case	
		1 linear flow of case	
Clarity	7	3 feedback	Feedback *“Sometimes it would say when I answered correctly and other times it didn’t. It was most helpful when I knew if I was right in my reasoning.”*
		1 answer choices	
		1 questions	
		1 spelling	
		1 grammar	
Quantity of Text	4	4 reduce the text	Reduce the Text *“Some pages of the VPS had a lot of text. Since we were given a time limit to get as far as we could in the case, we found that we would briefly skim or just completely skip these long passages.”*
Time Constraints	10	8 20 min insufficient	Time Constraints “*I would like more time for each activity in order to be able to absorb the materials more completely.”*
		2 timer	

^a^History of the Present Illness (HPI)

Table 9 summarizes the eight elements of engagement (as presented on the exit survey), and provides at least one other source of data for triangulation for each exit survey statement.

**Table 9:** Elements of engagement supported by VPS activities

Exit survey items related to engagement	% Strongly agree or agree	Supported by
1. VPS added variety to the learning environment.	92.4 %	Tutor comments
		Analysis memos
2. I enjoyed working on the tasks.	85.6 %	Photographs
		Tutor Feedback
		Analysis Memos
3. VPS provided exposure to new experiences.	78.8 %	Observations
4. VPS provided relevant feedback.	78.8 %	Observations
5. VPS increased my interest in clinical practice.	71.5 %	Photographs
6. I did not realize how time passed.	67.3 %	Photographs
		Analysis Memos
7. I found the tasks to be quite exciting.	63.5 %	Photographs
		Analysis Memos
8. I was completely absorbed in the activity.	60.6 %	Photographs
		Tutor Feedback

Taken together, these data indicate VPS foster engagement in eight dimensions.

### Design solutions for the next implementation of VPS

To answer the second research question, [How can these activities be improved next implementation?], student exit survey open responses were triangulated with observation notes and analysis memos and tutor feedback from the four VPS activities. Upon consideration of the residual issues reported from several data sources, design solutions for the next implementation of VPS are presented in Table 10.

**Table 10:** Design solutions for improving the next implementation

Issue category	Note	Design solution
Excessive Text	There is still too much text on the page, and there are extraneous review pages.	Streamline the cases
Time Constraints	20 min was not long enough to finish certain cases.	Provide the option to continue beyond 20 min.
Case Content	Medical content must fully align with content taught during the week in large group.	Continue to refine the cases to match specifics of large group content.
Case Format	Some of the VPS cases are still too lengthy and complex. Some of the feedback should be more specific.	Require cases be submitted months ahead for a review process. Ensure that they adhere to style guidelines.
Medical Record	Improve the patient history notes or provide students with access to the patient’s cumulative record.	Add a more realistic EMR that appears at regular intervals.
Activity format	When three triads are in one small group room, the sound of video media is too loud.	Student triads should meet in separate spaces or be able to listen via headphones.
Individual Play	Some students indicated that they want to complete the case individually.	Provide a library of cases on the learning management system (LMS) for individual practice after the peer-collaborative in-class activity.
Accessibility	Tutors expressed a wish for the VPS to be accessible through the LMS.	Integrate VPS with LMS.
Case Frequency	First year responses from a cohort receiving more than 20 VPS was less enthusiastic than those receiving only four VPS in the first semester. Tutors request a maximum of one VPS per class period, and not more than two per month.	These data suggest that about six VPS per semester might be acceptable to both faculty and students.

#### Excessive text

Both student exit survey and tutor feedback suggested the VPS cases sometimes contained too much text. In some cases, the amount of required case content reading impeded discussion. Prior to the second implementation, authors referred to case-writing guidelines and wrote more concise text. The VPS require additional revisions to further consolidate text without reducing the sophistication of the content. This same phenomenon was also reported by Bateman et al. [1].

#### Time constraints

The original lesson design was constrained by a time limit of 20 min, which was not always enough time for completing a VPS. These data suggest that in the next implementation, either the cases should be further streamlined, or tutors have the option to allow longer than 20 min for the VPS.

#### Case content

Students indicated case content should be tightly aligned to previous week’s large group lectures. During the data collection phase, VPS authors made an effort to align VPS case content to large group content, and in the fall of 2014, a more robust clinical faculty peer review system was implemented.

#### Individual play/accessibility

Some first year students indicated a preference for studying the cases at home. During this phase of implementation, cases were posted for a week after each implementation and students were encouraged to review the case individually at home. At the time of this writing, cases have now been set up in a course shell in the Blackboard LMS as a library, making them easier for students to access.

#### Case format

Observer memos corroborate that students held rich discussions about clinical cases, but struggled with difficult concepts. For example, students expressed a measure of confusion when they encountered imaging or lab choices that were challenging for them (especially given the 20-min time limit). One tutor wrote: *While the students show struggle and spend more than 10 extra minutes running to the end of the session, they show great interest in this case, and in obtaining the correct diagnosis.* Education research [55] indicates a measure of intellectual struggle is healthy and is especially associated with short term failure for complex or abstract tasks. For example, some observed student interactions included expressions of frustration and confusion in synthesizing and prioritizing investigations. However, after the VPS activities, students had an opportunity to debrief with tutors and clear up confusion.

#### Cognitive overload

Other student feedback suggested that solving a case at this pace resulted in a measure of cognitive overload [56]. Comments included excessive text, lack of a timer, too many *teaching pages*, ambient noise, and team members talking while others were reading. In the future, designers should streamline VPS to avoid the elements causing cognitive overload. In the field of primary care, it is common knowledge that fast thinking and intense data collection are required during patient encounters of less than 30 min. These training episodes build capacity to remain calm under pressure in a hectic, loud, collaborative clinic environment. Students were scaffolded by more knowledgeable peers and immediate feedback.

#### Medical record

Students requested the ability to go back in the case to check a patient’s chart or continuously refer to a patient’s medical record, as they would in authentic case practice. In the fall of 2014 new EMR forms were added to the VPS.

#### Activity format

Each case includes audio-visual media. While student and tutor comments reflected appreciation for these features, they could not play them loudly during group practice. Authors continued to pare down the duration of the videos as a result of this feedback, and consider devices such as splitters, so two or more students could listen to audio from one laptop.

#### Case frequency

Tutor feedback and student exit survey comments suggested it is best not to over-prescribe VPS during weekly case practice. Instead, VPS should be interspersed throughout courses, with a maximum of approximately six per semester.

## Discussion

To answer the research question regarding the ways VPS foster engagement, data were triangulated from researcher observations and memos, classroom photographs, tutor feedback and exit survey Likert ratings. In terms of the engagement sub-component flow, triangulation of findings between several data sources supported the assertion that VPS fostered flow. Survey data suggest that most students enjoyed activities, lost track of time, found activities exciting, or were absorbed in the activities. Analysis of 165 photographs during VPS indicated most students were focused when completing these exercises, an indicator of flow or absorption in task. Tutor feedback confirmed most students were involved and concentrating on the activity.

The second sub-component of engagement was interest. Triangulated findings support the assertion that VPS fostered interest. Exit survey results, researcher analysis memos and tutor comments corroborated that VPS, a new style of learning activity, seemed to engender enjoyment, focus, and humor, but also suggested elements of cognitive overload: excessive text, confusion, and noise generated from three students in the room. The third component studied was relevance. Taken together, exit survey results, observation analysis memos and screen captures support the assertion that VPS activities are relevant to student goals such as situational clinical practice, exam preparation and obtaining concrete feedback on clinical decisions.

That students elected to continue playing after their time expired was further evidence they enjoyed the VPS. Observations from the control room indicated that after completing the competency task, in nearly every case, even after the 20 min mark, if the case was not complete, students voluntarily returned to the case to finish it, and revealed their interest in more time by remarks on the exit survey.

Mixed methods findings for the eight dimensions of engagement (Table 9) lend support to similar assertions from game theorists, indicating that this generation benefits from a variety of teaching modalities in the learning environment [4, 5]. Literature from the field of situational learning [17, 57] indicates that students benefit from relevant feedback and exposure to new experiences. Feedback triangulated from Exit Survey responses, and Researcher observation memos provided input that were used to generate ten design solutions in order to improve the activities next implementation (Table 10).

## Conclusion

This study’s objective was to investigate whether VPS increase student engagement during clinical case practice. The triangulated evidence from multiple measures confirmed engagement in three dimensions: flow, interest and relevance. Photographic evidence and tutor observations confirmed that during a VPS, students were focused on the task at hand, an indication of flow [29]. VPS design features such as embedded movies, decision prompts, and score meters fostered opportunities to discuss the case. Exit survey results, observations, and tutor comments confirmed that the VPS provided variety and were relevant. Photographic data triangulated with tutor observations suggested a high level of student engagement, such as leaning in, avid peer discussion, humor, and the expressed desire to continue longer with the case.

Barriers to better engagement included time constraints, excessive text, classroom noise, frustration with difficult concepts and limited interaction with tutors. Photographs and tutor comments provide a different perspective; students appeared to be absorbed and focused on the activity. While the students were highly focused on the clinical tasks, survey responses and analysis of student dialog revealed that some parts of the clinical cases were challenging and students were concentrating on point totals under time duress. Grappling with challenging features of complex problem solving tasks is described in the literature as “struggle” [55]. However, in each VPS case observed, students pooled knowledge to reason through the evidence to arrive at consensus decisions. There were opportunities during a subsequent debrief with clinical tutors to clarify muddy points. Taken together, the body of data supports the goal-orientation aspect of flow theory, that students derive satisfaction from activities which relate to student goals [32]. They were motivated to finish tasks on time and provided detailed and abundant input on the exit survey.

### Limitations

The assertions from this study must be considered as naturalistic generalizations [10], meaning they are generalizable to the specific study context. While results from this study may be considered valid for the study site, they cannot be generalized to a wider population. These findings, supported by data triangulation, may be of use to other investigators in designing studies or in testing theories in new contexts. In attempting to apply these methods and findings toward an innovation in a different context, investigators should consider the specific constraints, type of VPS employed, outcome measures used, and the natural environment of the study setting.

### Future directions

Results from this study support continued integration of VPS in the curriculum, with attention to continuous quality improvement cycles as the cases are implemented in the classroom. Findings from this study provide evidence in support of specific design enhancements such as streamlining text, adding navigation tools, and refining the lesson plan to increase student engagement. This means limiting the number of VPS activities, keeping them streamlined and brief and allowing opportunities for students to interact with clinical tutors as much as possible. This research uncovered exciting potential for photographic data analysis. In the future we intend to continue to evolve the codes and categories in the photographic analysis rubric and conduct a rater reliability study [47]. During this implementation, VPS authors experimented with gamification elements adding a community healthcare thematic back drop, connecting characters from case to case and constructing care dilemmas with three different levels of patient outcome: a patient recovers fully, a patient requires surgery reducing quality of life, or a patient dies. Even though describing these designs in detail fell outside the scope of this paper, this research inspired new directions for dramatizing the cases to make them more engaging and realistic, with the ultimate goal of modeling a new paradigm of patient care that is team-based and patient-focused.

## Additional files

Additional file 1:
**VPS typology.** (DOCX 64 kb) Additional file 2:
**Observation form.** (DOCX 30 kb) Additional file 3:
**Tutor feedback form.** (DOCX 39 kb) Additional file 4:
**Exit survey.** (PDF 163 kb)

## Abbreviations

ATSU-SOMA: A.T. Still University’s School of osteopathic medicine in Arizona
HPI: history of the present illness
NMSk: neuro-musculoskeletal
TEAL-MEd: Technology-Enhanced Active Learning for Medical Education
VPS: virtual patient simulation
