# Supplementary material for: Evaluating medical student engagement during virtual patient simulations: a sequential, mixed methods study
Source: BMC Med Educ. 2016 Jan 16;16:20. doi: 10.1186/s12909-016-0530-7 (PMC4715308; doi:10.1186/s12909-016-0530-7)
Supplement: Additional file 1: — VPS typology. (DOCX 64 kb) [file 12909_2016_530_MOESM1_ESM.docx]

**Appendix D: A VPS Typology**

| **General** | Title | Virtual patient case practice in Decision Sim. Recently, these VPS have evolved to be part of the Virtual Community Health Center. |
| --- | --- | --- |
|  | Description | Free text |
|  | Language | English |
| **Educational** | Level | Designed to be adapted for use during all years of medical school. |
|  | Modes | Learning, formative assessment, deliberate practice |
|  | Coverage | Limb pain, regional back pain, seizure, dizziness |
|  | Objectives and outcomes | See Fig. 1 below. |
| **Instructional Design** | Path type | Branching design, using a gradual disclosure approach. Students plan an approach to a focused history and examination, based on the complaint |
|  | User modality | Students work in teams of 3-4. They discuss each clinical decision in the case. Alternatively, these VPS may be used for individual case practice. |
|  | Media & Resources | Use of images, audio, video. |
|  | Narrative Use and Patient Focus | These VPS are designed to provide a story line that prompts humanism and patient-centered approaches. There is a balance between presenting the data, and engaging with the patient. These VPS also emphasize cultural competency, osteopathic treatment, and teamwork. |
|  | Interactivity Use | During each episode, student teams make about 20 decisions, spanning all parts of the patient encounter. |
|  | Feedback  Use | Students receive prefabricated, written, immediate feedback about each decision throughout the case. |
| Technical | Originating system | Decision Simulation^TM^ |
| Format | Format | Text/HTML |
|  | Integration | System independent. |

Huwendiek et al. 2009, Med Teach. 2009 Aug;31(8):743-8.

| 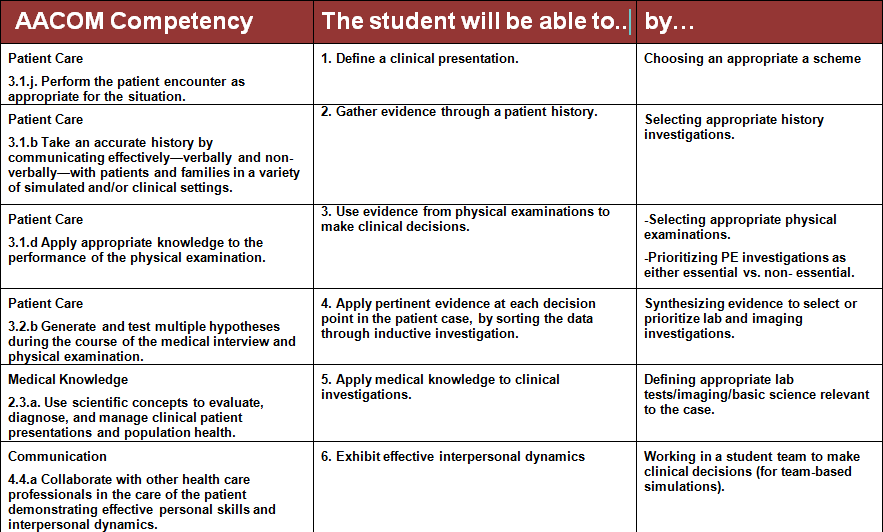 |
| --- |
| VPS competency areas aligned to national standards. Reprinted with permission. |
