# Supplementary figures and images for: Evaluating medical student engagement during virtual patient simulations: a sequential, mixed methods study
Source: BMC Med Educ. 2016 Jan 16;16:20. doi: 10.1186/s12909-016-0530-7 (PMC4715308; doi:10.1186/s12909-016-0530-7)

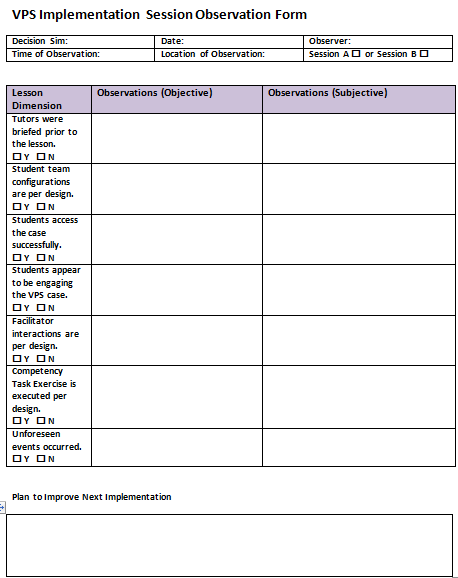

Supplement: Additional file 2: — Observation form. (DOCX 30 kb) [file 12909_2016_530_MOESM2_ESM.docx]

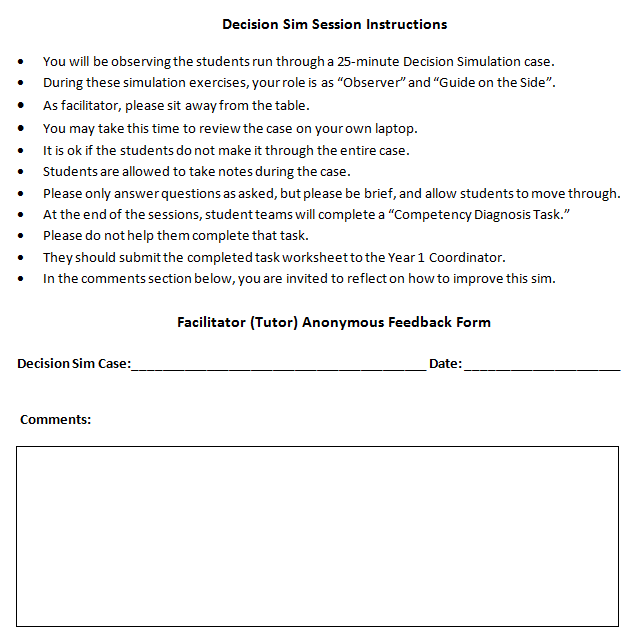

Supplement: Additional file 3: — Tutor feedback form. (DOCX 39 kb) [file 12909_2016_530_MOESM3_ESM.docx]
