# Supplementary material for: Evaluating medical student engagement during virtual patient simulations: a sequential, mixed methods study
Source: BMC Med Educ. 2016 Jan 16;16:20. doi: 10.1186/s12909-016-0530-7 (PMC4715308; doi:10.1186/s12909-016-0530-7)
Supplement: Additional file 4: — Exit survey. (PDF 163 kb) [file 12909_2016_530_MOESM4_ESM.pdf]

Fall 2013 VPS Exit Survey

Dear Students,

Thank you for providing feedback regarding Decision Simulation. Responses from this survey will remain anonymous, and will be used for educational research and the improvement of the curriculum. The researchers did not place any codes on the questionnaire that could directly identify you. There are no foreseeable risks or discomforts to you in SOMA's sharing the aggregate results of this educational research. Any publishable findings will be reported in aggregate format.

Completing this questionnaire should take 10 minutes. There are no direct benefits to you for completing this questionnaire. For questions about this research project, please contact Lise McCoy, Curriculum Specialist at lmccoy@atsu.edu.

1. I agree that my survey responses may be shared in anonymous format in research publications.

- ◯ Yes.
- ◯ No.

2. What is your age range?

- ◯ 20-25
- ◯ 26-30
- ◯ 31-35
- ◯ 36-40
- ◯ 41+

3. What is your gender?

- ◯ Male
- ◯ Female

4. Please indicate your learning preference in terms of echo-360.

- ◯ a) I always rely on Echo360 for lecture content instead of attending lectures.
- ◯ b) I mostly rely on Echo360 for lecture content and sometimes attend lectures.
- ◯ c) I sometimes rely on Echo360 for lecture content in addition to attending most lectures.
- ◯ d) I rarely rely on Echo360 for lecture content since I regularly attend lectures.

Other (please specify)

[ ]

Fall 2013 VPS Exit Survey

5. INTEREST: Please rate the value of the virtual patient simulation activities in terms of interest and relevance.

	Strongly Agree	Agree	Neutral	Disagree	Strongly Disagree
1. They increased my interest in clinical practice.	◯	◯	◯	◯	◯
2. They added variety to the learning environment.	◯	◯	◯	◯	◯
3. They provided relevant feedback.	◯	◯	◯	◯	◯
4. They provided exposure to new experiences.	◯	◯	◯	◯	◯

6. FLOW: Please rate the value of the virtual patient simulation activities in terms of whether you were absorbed in the task.

	Strongly Agree	Agree	Neutral	Disagree	Strongly Disagree
I did not realize how time passed.	◯	◯	◯	◯	◯
I enjoyed working on the tasks.	◯	◯	◯	◯	◯
I was completely absorbed in the activity.	◯	◯	◯	◯	◯
I found the tasks to be quite exciting.	◯	◯	◯	◯	◯

7. CLINICAL DECISION MAKING. Please rate the critical thinking aspects of the virtual patient simulation activities.

	Strongly Agree	Agree	Neutral	Disagree	Strongly Disagree
1. They provided practice with schemes and inductive reasoning.	○	○	○	○	○
2. They increased my evidence sorting abilities.	○	○	○	○	○
3. They helped me review for exams.	○	○	○	○	○
4. They integrated theory with practice.	○	○	○	○	○
5. During VPS activities, I made decisions about the sequence of the patient encounter.	○	○	○	○	○
6. During VPS activities, I gathered evidence from physical examinations to make clinical decisions.	○	○	○	○	○
7. During VPS activities, I synthesized evidence to prioritize lab and imaging investigations.	○	○	○	○	○
8. During VPS activities, I applied pertinent evidence at each decision point to reason toward a ballpark diagnosis.	○	○	○	○	○

8. COLLABORATION. Please rate the social-collaborative aspects of the virtual patient simulation activities.

	Strongly Agree	Agree	Neutral	Disagree	Strongly Disagree
1. Brain-storming with fellow medical students was helpful.	○	○	○	○	○
2. Team discussion clarified concepts.	○	○	○	○	○
3. Group decision making was useful.	○	○	○	○	○
4. Working in a small team of 3 allowed better participation than working in a group of 10.	○	○	○	○	○
5. I communicated in a professional manner using respectful language.	○	○	○	○	○
6. I encouraged other members on the team to express their opinions.	○	○	○	○	○
7. Other group members communicated respectfully with me.	○	○	○	○	○
8. I put in a lot of effort.	○	○	○	○	○

9. How can we improve these activities?
